# A Facial Attractiveness Account of Gender Asymmetries in Interracial Marriage

**DOI:** 10.1371/journal.pone.0031703

**Published:** 2012-02-09

**Authors:** Michael B. Lewis

**Affiliations:** School of Psychology, Cardiff University, Cardiff, United Kingdom; Université de Toulouse, France

## Abstract

**Background:**

In the US and UK, more Black men are married to White women than vice versa and there are more White men married to Asian women than vice versa. Models of interracial marriage, based on the exchange of racial status for other capital, cannot explain these asymmetries. A new explanation is offered based on the relative perceived facial attractiveness of the different race-by-gender groups.

**Method and Findings:**

This explanation was tested using a survey of perceived facial attractiveness. This found that Black males are perceived as more attractive than White or East Asian males whereas among females, it is the East Asians that are perceived as most attractive on average.

**Conclusions:**

Incorporating these attractiveness patterns into the model of marriage decisions produces asymmetries in interracial marriage similar to those in the observed data in terms of direction and relative size. This model does not require differences in status between races nor different strategies based on gender. Predictions are also generated regarding the relative attractiveness of those engaging in interracial marriage.

## Introduction

The majority of marriages in the US and the UK are between people of the same racial background (race is used here to indicate a broad group of ethnicities as employed in the US census). The incidence of interracial marriage, however, is increasing particularly in the US since the repeal of the anti-miscegenation laws in 1967 [1]. In the US, over 4% of marriages can be classified as mixed race (source: US Census Bureau, 2006). In the UK the figure is more like 2% (source: Census UK, 2001). Of particular interest here are the clear patterns that emerge from the analysis of which interracial marriages occur more often than others.

A striking aspect of the data on interracial marriages is the size of the gender asymmetries [1]–[3]. These asymmetries appear robust across time and culture. Details of these asymmetries are shown in Table 1 based on census data from the UK and USA for White, Black and Asian racial groups. If we focus upon marriages between White and Black people then we observe that there are over twice as many marriages between Black men and White women than between White men and Black women in the US. An observed consequence of this pattern is a decline in marriage rates for Black women, which has been described in the US as the ‘marriage squeeze’ [4]. The asymmetry is smaller in the UK but still present.

**Table 1:** Measuring the size of the gender asymmetries in interracial marriage.

*X*	*Y*	Percentage of *X* males marrying *Y* females	Percentage of *X* females marrying *Y* males	Size of asymmetry (largest divided by smallest)	Average asymmetry (from both complimentary measures)
**UK (Source: Census UK, 2001)**					
Black	White	17.60	13.27	1.32	1.46 Black Male Bias
White	Black	0.15	0.24	1.60	
White	Chinese	0.11	0.04	2.75	2.60 White Male Bias
Chinese	White	9.57	23.47	2.45	
Black	Chinese	0.115	0.05	2.30	2.30 Black Male Bias
Chinese	Black	0.14	0.32	2.29	
**USA (Source: US Census, 2006)**					
Black	White	6.61	2.85	2.32	2.38 Black Male Bias
White	Black	0.23	0.56	2.43	
White	Asian	1.03	0.34	3.03	2.84 White Male Bias
Asian	White	6.48	17.11	2.64	
Black	Asian	0.79	0.15	5.27	5.14 Black Male Bias
Asian	Black	0.22	1.10	5.00	

Each asymmetry is shown as a function of total marriages for each race involved before an average is found. Summary data were taken from Belot and Fidrmuc (2009).

The gender asymmetries are even larger for marriages that include Asian and White people. In this situation, however, it is the number of White men marrying Asian women that is over twice the number of White women marrying Asian men. The largest asymmetry shows that marriages between Black men and Asian women in the US outnumber those between Asian men and Black women by about five to one.

The current paper aims to explain the observed patterns of gender asymmetry in interracial marriage. First, existing accounts for the phenomenon are considered. One such account is that there are differences in societal pressures for males and females. Economics-based marriage models are considered but these require different statuses for different races and it is argued that they fail to capture the details of asymmetries. An explanation based on height differences is also explored but it is shown through data modelling how this can only explain part of the observed asymmetries. Finally, a new explanation based on facial attractiveness differentials between races for different genders is explored. For this to explain the patterns of asymmetries observed, however, a particular pattern of facial attractiveness must be present among the different races. An experiment is reported that acquired the necessary facial attractiveness data to explore this model further. From these data, the model was implemented in order to test whether it could explain the patterns of gender asymmetry observed in interracial marriage. A speculative evolutionary account is also provided as to why it is the case that differences in the perceived attractiveness of genders of different races occur.

### Societal pressures

One possible explanation for gender asymmetries in interracial marriage is that the there are differential societal pressures upon the different genders to marry within their ethnic group. A suggested example might be that males from the Indian sub-continent living in the UK might be freer to interact with the White community than females from the same community [5].

While there probably are some societal pressures acting against the formation of interracial marriages, this explanation for the observed asymmetries only works if these act differently upon male and female members of the same racial group. It has been found, however, that there is no evidence of differential societal pressures on East-Asian or Black men and women regarding interracial marriage [6]. These racial groups, together with White people, form the focus of analysis here and so societal pressures do not explain the patterns of data seen.

### Modelling interracial marriage

Models of marriage choice tend to see partner selection as operating within a ‘marriage market’ such that it improves each party's situation. Social-exchange theory of marriage proposes that there can be a trade off between one party's economic wealth and the other's status [7]. This theory has been used to explain why a rich but less physically attractive person might marry an attractive but poorer person [8]. Social exchange theory is used to explain why people tend to marry people who are similar in terms of their educational and/or socioeconomic background [9].

In relation to interracial marriage, race is often described as a marker of status in the marriage marketplace and examples are quoted where a wealthy Black man might marry a poorer White woman [10]. Based on this theory, it has been argued that interracial marriage occurs primarily were the White woman ‘marries up’ in socioeconomic status [11]. The claim is that the social exchange that takes place is between the woman's racial status for the man's socioeconomic status or wealth. As men may be economically more mobile than women, then this could be used to explain the gender asymmetries in Black/White interracial marriage.

This idea of race as being a status factor in the social exchange of marriage has been explored empirically [12]. It has been suggested that when people are presented with mixed-race couples, they are more willing to accept a Black man with an unattractive White woman than a Black man with an attractive White woman. It was concluded from this that people felt more comfortable when the low status man (arguably the Black man rather than a White man) was paired with the low status woman (arguably the less attractive woman). It was argued that this experiment supports the notion that racial status has objective value within the marriage market and there is a clear racial hierarchy with White people above Black people. The results of this study, and hence the conclusions, are limited by a number of facts: only White participants were tested on their opinions; only a Black male was used as the male partner, and the Black male in the experiment did not vary in attractiveness. The results, therefore only tell us about the hierarchy of racial status as perceived by White people when looking at Black males. Relationships between Black females and White males were not assessed and opinions of Black participants were not assessed.

Incorporating race as status into the social exchange theory of marriage is problematic. The origins of this social-exchange theory of marriage stem from caste systems of India [13]. In this system there is an agreed hierarchy between the different castes. This system does not translate easily to the American or British society in which there is no clearly defined hierarchy of ethnicities. Members of ethnic or racial groups would not consider their group to have legitimately a lower status than any other group (or else there would not have been the African-American Civil Rights Movement). Explaining interracial marriage in the US or UK in terms of social exchange, where one person's White status is exchanged for wealth or security, can be argued to be a White-centric myth. Research supports this social exchange to be a myth because interracial marriages show the same degree of similarity between partners' status as same-race marriages [14]. Hence, there is no evidence for racial status to be a commodity for social exchange in these cultures.

While it is accepted that there may be social exchange in marriage, it is argued here that race does not need to enter into this exchange in a hierarchical manner. As will be shown below, the gender asymmetries in interracial marriage can be explained without there being a racial hierarchy.

Another model of interracial marriage is the equilibrium sorting explanation [15]. Applying game theory, people select mates such that they maximise their productivity and, through equilibrium sorting, they maximise the productivity of the system. On this model, there is a cost associated with interracial marriage, but this can be outweighed by the gains of having a partner with high human capital (e.g., wealth or potential for wealth). A consequence of this is that individuals who choose to marry outside of their race will, on average, be more highly educated [1]. While this model is useful for explaining some of the data, there are two problems with this model. First, the data from education levels do not show interracial marriage to be more common among more educated people either in the US [14] or the UK [16]. Second, as the cost of interracial marriage applies across men and women, it does not explain the large asymmetries observed for interracial marriages. As we will see later, however, a variation of this explanation is able to capture the observed patterns of interracial marriage if we incorporate facial attractiveness into the model.

### Is it just height differences?

In spite of the decades of modelling of marriage data, none of the traditional economic models deal unequivocally with the issue of gender asymmetry in interracial marriage. One recent proposal, however, has been offered that does provide a possible explanation. This explanation is simple, elegant and is based on differences in the human anatomy between the races concerned.

It has been proposed that the gender asymmetries in interracial marriage can be attributed to differences in average heights of the race-by-gender groups [6]. It has been observed that Blacks, on average, are taller than Asians (based on the health survey for England, 2004) and this may affect mate choice. It is proposed that there is a socially imposed ‘male-superior norm’ such that the male should not be shorter than the female in a marriage and this factor alone can affect the patterns of intermarriage. For White females, this norm will not have much of an effect on their choice of White or Black partners, but, as Asian men tend to be shorter, the male-superior norm will reduce the number of potential Asian partners. This means that, all other things being equal, height will discount more potential Asian partners than either Black or White partners. There would, therefore, be a bias against White women marrying Asian Men that is not present for Asian women marrying White men. The same norm could also explain the Black/White asymmetry if Black women were taller than White women. The consequence would be that height would act to discount more Black than White women as potential partners for White men leading to the observed asymmetry.

Although this is an elegant explanation, there are limitations to how well it can explain the gender asymmetry in terms of height alone. There is little difference in the height of Black and White males or Black and White females and even the difference between heights between White and Asian people cannot explain all of the asymmetry. This can be demonstrated using Monte Carlo style analysis of population patterns.

To show the limitation of the height explanation, statistical modelling of the height data from the Health Survey for England (2004) was carried out. Random pairs of males and females were generated according to their height distributions for Black, White and Chinese people. Comparing these random pairs found few occasions when the women is taller than the man. Table 2 shows that the woman being taller than the man does occur more often when looking at Chinese men paired with White women: In this case, 18% of pairings would violate the male-superior norm. Given that in the UK there are two and a half times as many White males marrying Chinese females than the other way around, a reduction of 18% cannot entirely explain this pattern.

**Table 2:** Explaining the interracial marriage gender asymmetries using height.

	White Male 175.3 cm (7.3)	Black Male 174.4 cm (7.2)	Chinese Male 170.8 cm (7.4)
**White Female 161.6 cm (6.8)**	8.6%	10.0%	18.1%
**Black Female 162.9 cm (6.6)**	10.4%	12.0%	21.4%
**Chinese Female 157.9 cm (6.0)**	3.3%	4.0%	8.8%

The table headings show the average heights (and standard deviations) for the difference racial-by-gender groups. The entries in the table show an estimated percentage of pairings that would result in the male being shorter than the female, hence violating the proposed male-superiority norm.

The comparison between the Black and White pairings is also difficult to reconcile with the observed data. A typical White man is shorter than a typical Black woman 10.4% of the time whereas a typical Black man is shorter than a typical White woman 10.0% of the time. In this case, the male-superior norm can only explain a tiny proportion of the gender asymmetry observed in intermarriage between Black and White people.

While difference in height between the different races can explain some of the observed gender asymmetry in interracial marriage, it does not explain the strength of the patterns observed. Height may certainly have a role to play but there must be other factors also contributing to interracial partner choice patterns.

### An explanation based on facial attractiveness

There exists a lay understanding that choosing who we marry is related to physical attraction. This relationship is supported by psychological research into physical attraction on mate selection particularly with reference to identifying good genes [17]. There is clear and unequivocal evidence that physical attractiveness is the primary mating asset for women such that attractive women are preferred over unattractive women [18]. For men however, status is an important mating asset although physical attractiveness can still carry some weight [19]. Much of the evidence for the differences in preferences between men and women, however, comes from self reports and reflections rather than actual preferences at the point of marriage. Where marriage couples are asked about their important considerations in marriage partners, terms such as romantic love and a desire to set up home are more important and there is little difference between the sexes [20]. In fact, men and women may be behaving very similarly in terms of their marriage partner selection.

The focus here is facial attractiveness of both the males and females. Facial attractiveness of a person is indicated by the rated attractiveness of a person from a portrait. A person's facial attractiveness is typically the first judgement that another person makes of them from which it can be judged whether they are likely to ultimately enter into a relationship with them. These kinds of portraits are widely used by dating agencies as a method for people to select potential partners and so have face validity in terms of being used to select marriage partners.

Facial attractiveness is not necessarily the same as physical attractiveness. The latter may include measures of bodily attractiveness such a waist-hip-ratio for women. Further, facial attractiveness, as derived from a natural portrait, may contain status information or information about the person's personality or at least the personality the person wishes to portray. In this way, facial attractiveness appears to capture elements of the reported preferences for both males and females.

Facial attractiveness receives little attention in models of the marriage market in favour of more tangible assets. Here, it is proposed that measurable facial attractiveness differences between different races can be used to explain the interracial marriage gender asymmetry. Further, it can do so without treating males and females differently and without enforcing a racial hierarchy.

Studies suggest that there is considerable agreement regarding what makes a face attractive [21]. Much of this agreement is common even across cultures [22]. Further, just as not all races are equal in terms of their average height, not all races of people are equal in terms of their average rated facial attractiveness. Such differences may affect any model of marriage but here a simple model is presented in order to further investigate the effects that differences in attractiveness might have.

The model of marriage proposed here is based upon contact, cost and chance. The first principle is that people tend to marry people that they come into contact with. The degree of separation between races, therefore, explains why the majority of marriages are intraracial. This contact principle also accounts for why married couples tend to have a similar economic status or educational background to each other [14] as such people are more likely to come into contact with each other. The second principle is that, although marriage is desirable, there is a degree of cost associated with any marriage. There are two parts to this cost: First there is the exclusivity of the relationship meaning that other marriages are no longer possible (at least in the short term). The size of this cost will be a factor of the attractiveness of the potential partner such that the cost is lower if the potential partner is more attractive as there will be fewer more attractive partners that the person will be missing out on. The second part of the cost comes from the racial or ethnic difference between the potential partners. This is similar to the cost in the equilibrium sorting model and is related to the degree of dissimilarity between the racial or ethnic backgrounds of the two potential partners. This racial difference cost will be related to the acceptability of the racial pairing for that culture. This cost principle can account for the increasing trend in interracial marriage in the US during the latter part of the twentieth century as racial distance decreases [23]. The final principle is that there is an element of chance in any pairing becoming a married couple. That is, given that two people have come into contact, there is a chance that they will get married and this is a probabilistic function influenced by the cost of that marriage to each partner.

One observation about this model is that racial distance is always symmetrical and is not affected by gender. The racial distance will be the same regardless of whether a Black man is paired with a White women or a White man is pair with a Black women. In this way, it overcomes the problems of the social-exchange theory in which a particular hierarchy of races is required because all races and both genders have equal status. The implementations of the model reported here also used a fixed cost for all interracial marriage regardless of which racial boundaries are crossed. In the general form of the model, the cost could be related to how dissimilar the racial groups are.

A second observation is that a person's own attractiveness does not affect their decision to marry another person. A consequence of this is that an attractive person paired with an unattractive person will be more likely to marry than two unattractive people. From the point of view of the attractive person, however, they will still be more likely to marry an attractive person given the probabilistic nature of the chance part of the model. Unattractive people will still be able to marry but it would require more pairings, each pairing having a particular probability of success – albeit, a probability that would always be higher if they were more attractive. In this way, a degree of attractiveness sorting would take place. Evaluations of the marriage photographs shows that the correlation between the attractiveness of married couples is around *r* = 0.34 [24] and below it is demonstrated how the model predicts similar correlations.

Importantly, the model can account for the gender asymmetries because those individuals who are more attractive are more likely to be able to overcome the cost associated with interracial marriage. If there are differences between the relative attractiveness of the genders between different races then asymmetries in interracial marriage will follow. If Black men are perceived as being more attractive than White men and White women are perceived as more attractive than Black women then the type of asymmetry observed in interracial marriages would be a direct consequence of the model. Of course, the explanation only works if the pattern of attractiveness is as described.

Data from previous studies support the required pattern of facial attractiveness over different racial groups. Black men were rated as being significantly more attractive than White men; however, little difference was found between women [25]. A follow-up study found that White women were rated as more attractive than Black women although this was not significant once a conservative Bonferroni correction had been applied [26]. Further experimentation is required, therefore, to clarify these findings.

The model of gender asymmetries in interracial marriages can also be applied to marriages involving Asians as well as Black and White people. The asymmetry here is that there are more female Asians than male Asians involved in the interracial marriages. This could be explained if it transpired that female Asians were more attractive than female Black or female White people on average and if male Asians were less attractive than Black or White males. This kind of data does not currently exist (although one study did show a difference in attractiveness but this was based on a single female example of each racial group [27]). If the attractiveness explanation for gender asymmetries is to stand, then it is necessary to determine whether there really are differences between average attractiveness for people of different races.

## Methods

The current experiment aimed to establish the relative attractiveness of individuals of three broad racial groups. These attractiveness ratings were made by people of a similar age to the individuals and of an opposite sex. The raters came from a range of different ethnicities and any differences in their ratings of different races were considered separately.

### Ethics

The research was approved by Cardiff University School of Psychology research ethics committee. Informed written consent was obtained from all participants.

### Participants

Forty undergraduates studying at Cardiff University took part as face raters either for course credit or for a small cash payment. Twenty were female and twenty were male and all were between the ages of 18 and 30 years. Of the male raters, 15 were White, 2 were Black and 3 were Asian. Of the female raters, 14 were White, 3 were Black and 3 were Asian.

### Stimuli

Images of 600 people were used. Half were male. The images were collected from the social-networking website *Facebook.com*. Images were selected from people who were members of groups associated with further and higher education bodies either in the UK (for White faces), sub-Saharan Africa (for Black faces) and East Asia (for Asian faces). These images were collected by a naive research assistant who selected images according to a set of criteria: Images had to show a clear view of a single person that was of sufficient quality such that it would be recognisable by a friend. If the face in the image had a weird expression or was possibly of a race other than the main race for the region then it was rejected. The face was also rejected if the person depicted looked to be under 18 or over 30 years old.

This method of stimulus generation was employed as being the best of those available to produce a representative set from each population. By using images that the individual freely posted on the internet to represent themselves, we avoid many of the problems associated with self selection had we used standardised photographs: that is, individuals who are particularly self conscious about their appearance will not volunteer. Selection bias is likely to be less true for facebook images as posting an image of oneself is what everyone else is doing. There would, of course, be a bias to select a photograph that presents one's most positive image but this bias would be present across all races and genders. One might assume that if these people were to use online dating websites then they would use an image similar to their facebook image in their profile. As such, this means that the stimuli employed are similar to the information presented during courtship, which is appropriate as marriage is the focus of the research.

One potential problem with this set of stimuli is the possibility that one group might be more reluctant to post images of themselves if they are less attractive than another. If this were the case then we would expect to see more facebook images that do not contain a face of the person (it might be left blank, be a scene or a cartoon character). Re-examination of the sources of the images used revealed that less than 1% of facebook accounts did not include a face of a person.

### Procedure

Participants were presented with 300 opposite-sex faces, one at a time via a computer monitor. They rated each of these faces on their attractiveness. The faces were presented in a random order and the rating scale went from 1 (unattractive) to 10 (attractive).

## Results

The responses of all participants to all of the faces are available as a supplementary file called Data S1. Table 3 shows the summary means and standard deviations for the sets of faces. For the female faces, Asian faces were rated as being most attractive followed by White and then Black. A three-way ANOVA showed these differences to be significant (*F*(2,297) = 11.861; *p*<.001) with all of the comparisons significant (*p*'s<0.05). For the male faces, Black faces were rated as being most attractive followed by White and then Asian. A three-way ANOVA showed these differences to be significant (*F*(2,297) = 63.305; *p*<.001) with all of the comparisons significant (*p*'s<0.05).

**Table 3:** Findings from the current research.

	Male faces rated by females	Female faces rated by males
**White**	4.568 (0.869)	5.065 (1.347)
**Black**	4.994 (0.798)	4.720 (0.732)
**Asian**	3.781 (0.653)	5.511 (1.104)

The means (and standard deviations) for the attractiveness ratings for the sets of 100 faces from each group. The scale ranged from 1 (unattractive) to 10 (attractive).

**Figure 1:**
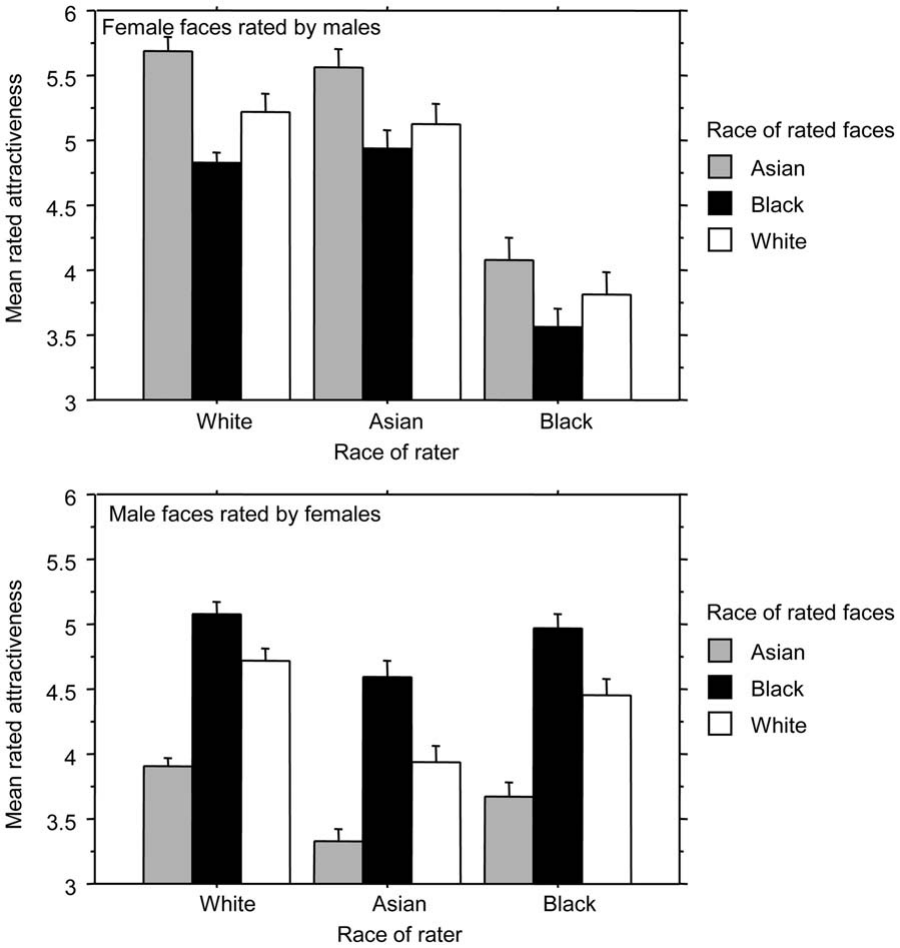
Findings from the current research. Patterns of perceived attractiveness ratings for faces of different races and different genders split according to ratings by participants of different races. Error bars show standard errors by faces.

It may be the case that the ethnicity of the raters influenced their ratings. This was investigated using a by-subjects analysis. Figure 1 shows that the same ordinal pattern was found when the data were split according to the participants' ethnicity. Two ANOVAs were conducted on the data sets in order to explore the possibility of there being an interaction between race of the rater and race of the face being rated. These interactions were not significant (female faces: *F*(4,34) = .403; *p*>.05, male faces: *F*(4,34) = .175; *p*>.05).

## Discussion

The results replicate earlier findings that Black men are rated as more attractive than White men. It was further found that Asian men were rated as less attractive than either other race. For women the pattern was reversed with Asian women being rated as most attractive followed by White women and then Black women. The patterns observed occurred regardless of the race of the person doing the ratings.

It is argued here that this pattern of attractive ratings is sufficient to explain the gender asymmetries in interracial marriage. In order to explicitly explain this argument, a model of marriage based on attractiveness was tested using the attractiveness data acquired here.

### Data modelling

An implementation of the attractiveness-based marriage model was carried out in which the 10,000 individuals (half female and half male) were randomly assigned to being Black, Asian or White. The attractiveness of each group of individuals was randomised such that they had the same mean and standard deviation as observed in the experiment above.

During an iteration of the model, a random unmarried male and a random unmarried female were selected. If these were of different races then a racial distance value was subtracted from their attractiveness to indicate the cost of crossing racial boundaries. The probabilistic function association with the chance element of the model was implemented by subtracting the attractiveness of another random unmarried person of the same gender from the resulting values. If the resulting values were greater than some arbitrary threshold for both the male and the female then the marriage was considered to take place. A new pair of individuals would then be considered in the same way for the next iteration. The model was iterated until 90% of the individuals were married. At this point the model was assessed as to how many interracial marriages had occurred and what patterns were more common when they did occur. From this information, the asymmetries of interracial marriages were measured for the model.

The model was implemented several times with varying values taken for the threshold (varying between 0 and 2) and the racial distance (varying between 0.01 and 2). The parameters were optimised to account for the patterns of asymmetries found in the UK population. A threshold of 0.65 and a racial distance of 0.65 gave the following pattern: the Black/White asymmetry was 1.41; the White/Asian asymmetry was 2.59, and the Black/Asian asymmetry was 2.35. These figures compare well with those in Table 1 for actual asymmetry in the UK.

The parameters of the model were also optimised to account for the patterns of asymmetries found in the US population. A threshold of 0.45 and a racial distance of 1.00 gave the following pattern: the Black/White asymmetry was 1.67; the White/Asian asymmetry was 3.57, and the Black/Asian asymmetry was 5.41. While these numbers are similar in relative order and approximate size to those in Table 1 for US data, there remains considerable difference that could not be reduced by further changes to the parameters of the model.

There are at least three possible reasons why the model fits the UK data better than the US data. First, the ratings used were from people based in the UK and there could be cultural differences in ratings between the UK and the US. Second, the US values in Table 1 are calculated using figures for all Asians whereas the data used in the model was based on East-Asians. Third, many of the Black people that make up the samples in the US data may have been mixed-race (the US census does not offer a mixed-race category). It has already been demonstrated that mixed race people are rated as more attractive than Black or White people [25] and so this could affect the fit of the model. Regardless of the mismatch between the model and the US data, it remains the case that a model of marriage based just on race and attractiveness is able to capture the general patterns of gender asymmetry in interracial marriage observed in the US.

The model could be further interrogated with regards to the nature of the pairs in made. The correlation between the attractiveness of the pairs of partners put together by the model was found for the different implementations. An implementation with no racial differences (and a threshold set to zero) found a correlation of *r* = 0.32. In the model of the UK data, it was *r* = 0.49 whereas for the model of the US data it was *r* = 0.40. These are all similar in scale to the actual correlation (*r* = 0.34) observed in wedding photographs [24] and therefore the model shows a degree of attractiveness sorting.

**Figure 2:**
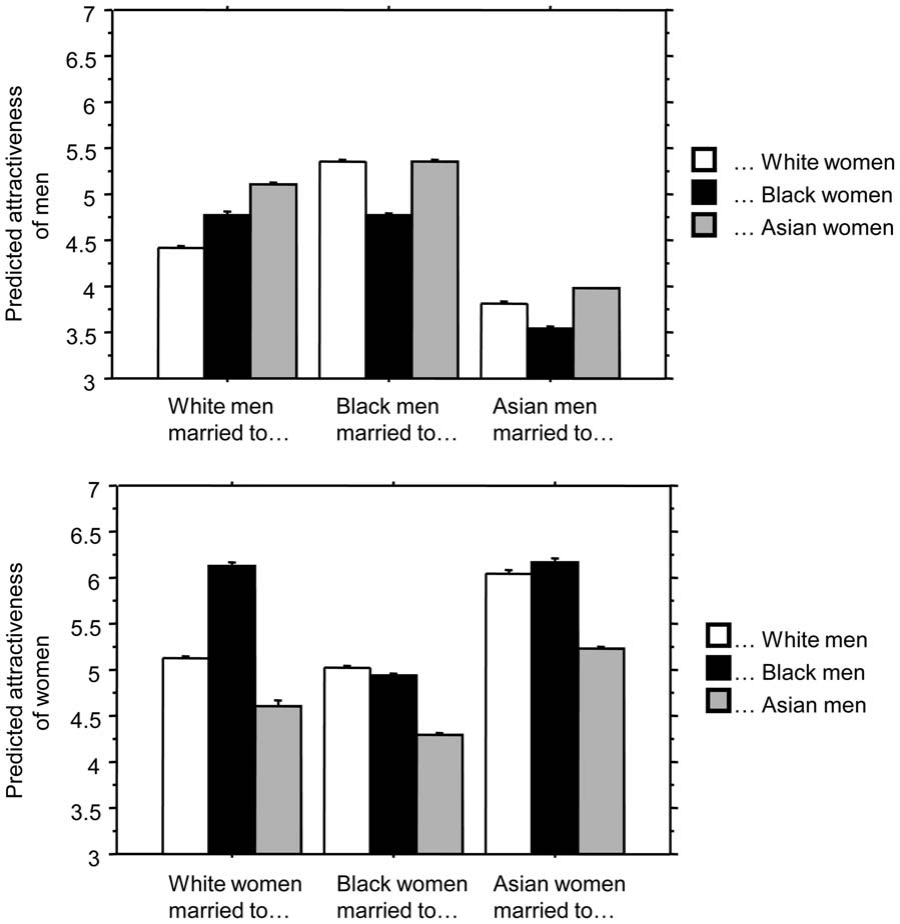
Predictions of the marriage model regarding the mean attractiveness of groups involved in marriage to same or different ethnic groups.

The pattern of attractiveness seen in mixed-race and non-mixed-race couples also leads to a series of predictions for this model. These predictions come from finding the relative attractiveness, within each group, of those engaging in mixed-race or same-race partnerships (see Figure 2). For White and Black men, it was observed in the model that those engaged in mixed-race couples tend to be more attractive that those engaged in same-race couples, whereas for Asian men, the more attractive men married same-race women. For White and Asian women, it was observed that those engaged in mixed-race couples tend to be more attractive than those in same-race couples (except for White women marrying Black men). Finally, Black women engaged in same-race couples tended to be more attractive than Black women married to Asian men. It is a prediction of the model, therefore, that similar patterns will be observed in attractiveness patterns of real couples. This will be the focus of future research as such patterns could be assessed by obtaining ratings for individuals in wedding photographs published in newspapers.

### Why do the differences exist?

So far it has been shown that the patterns of perceived attractiveness for people of different races are different for males and females. It was also demonstrated how these differences could explain observed patterns of gender asymmetries in interracial marriage. What is not explained is why the different patterns of attractiveness occur for different races. Here, some speculative evolutionary ideas are reviewed that can explain the patterns.

First, it has been demonstrated that skin colour is a sexually dimorphic characteristic. Men tend to have darker skin than women [28], [29]. Further, in the majority of cultures that have been tested, there is a bias that lighter skin pigmentation is considered more attractive in women [28]. This sexual dimorphism can explain why Black men and White women are considered more attractive than White men and Black women respectively. The former represent highly positive sexually dimorphic patterns. This does not, however, explain the findings with regards to Asian attractiveness measures as their skin tones tend to be between those of Black and White people.

In order to provide a possible explanation for the pattern of attractiveness for Asian people, one can look to the evolutionary impact of the environment in which the races developed. Frost hypothesised that many of the visual features that distinguish White from Black people are a result of differences in patterns of sexual selection [30]. Further from the equator (for example in the arctic tundra of Europe 10,000 years ago), men would be less available for two reasons. First, they would have to hunt over greater distances with increased mortality. Second, polygamy would be less common due to having to provide over a longer winter. As a result, away from the equator, there would be greater competition between women for mates. This competition would lead to sexual selection for more feminine characteristics. While the sexual selection would be driven by competition between females, it would act upon both the males and females making them both more feminine. At the same time in the agricultural parts of Africa, females could contribute more to food production and so could be more easily supported. Men would be able to take more than one wife and so women would be competed for by males. Competition between males for mates would lead to sexual selection of masculine traits. Again, these traits would carry over into both the males and the females. This pattern of evolutionary development, therefore, provides an explanation for why White females and Black males are perceived to be more attractive than Black females and White males.

Although not specifically considered by Frost, this geographic evolutionary explanation can be extended to explain the findings regarding the perception of attractiveness of Asian people as well. We can do this if we assume that, just like the arctic tundra conditions of Europe, the mountainous expanses of Asian lead to a lifestyle of difficult agriculture. Several males may be required to support a single female as is currently the practice in the polyandrous Tibetan culture [31]. Such a society would show sexual selection for feminine features as a highly feminine female would be able to attract the support during child rearing of one, or more, productive partners. In this case, however, it is not the lighter skin tones and fairer hair that were selected for but the rounder, more feminine face structure. In this way, competition between females for mates leads to a population that is more feminine in its facial characteristics. These feminine facial characteristics mean that Asian women are perceived as being more attractive whereas the same features affect the attractiveness of Asian men in a negative manner.

### Conclusion

The results of the experiment demonstrated that there are robust differences in the relative perceived attractiveness of different racial groups. Further, these differences are affected by the gender of the person being rated. Among males, Black faces were rated as the most attractive followed by White faces and then Asian faces. For the females, Asian faces were seen as the most attractive followed by White and then Black faces. The same pattern was found regardless of the ethnicity of the person doing the ratings.

A model of marriage is put forward in which facial attractiveness and race affect whether or not a couple marry. Facial attractiveness increases the chance of marriage whereas a difference in racial background will decrease the chance. It follows from this model that differences in patterns of interracial marriage will be a consequence of differences in average attractiveness of the gender-by-race groups: In general, more attractive the person is the more likely they are to be involved in an interracial marriage. Black men and Asian women (the most attractive groups) occur within interracial marriages more often than Asian men and Black women (the least attractive groups).

The results and model presented here represent a significant advancement in understanding the gender asymmetries in interracial marriage. Previous explanations have required a social-exchange of racial status [7], which implied a hierarchy of races. The current model of interracial marriage does not require this hierarchy but treats all races as equal except in terms of subjective ratings of attractiveness. Further, unlike the equilibrium sorting explanation [15], the attractiveness account does not predict that people in interracial marriages will be better educated than those not, although it does predict differences in their attractiveness. Finally, there may be a role for differences in height to play in marriage particularly as the perceived attractiveness of males may be related to their height, but the data and analysis presented here tells us that rated attractiveness alone can account for the patterns of data observed.

It is clear that physical attractiveness is not the only feature that people use in making a decision about the person they marry. The research reported here, however, indicates that attractiveness patterns across different races are sufficient to account for why such large gender asymmetries exist when people of various races marry.

## Supporting Information

Data S1 **The raw attractiveness responses for the 40 participants for the 600 faces.** The information includes the race of the raters and the race of the faces being rated.
(XLSX)
